# Markers for the Severity of Multisystem Inflammatory Syndrome in Children: A Multivariate Analysis

**DOI:** 10.1155/ijpe/5409585

**Published:** 2026-04-10

**Authors:** Georgi Vasilev, Nadzhie Gorelyova, Russka Shumnalieva, Latchezar Tomov, Dayana Gandi Abousaid, Iren Tzotcheva, Snezhina Lazova, Tsvetelina Velikova

**Affiliations:** ^1^ Laboratory of Clinical Immunology, National Specialized Hospital for Active Treatment of Hematological Diseases, Sofia, Bulgaria; ^2^ Medical Faculty, Sofia University “St. Kliment Ohridski”, Sofia, Bulgaria, uni-sofia.bg; ^3^ Pediatric Clinic, University Multiprofessional Hospital for Active Treatment and Emergency Medicine “N. I. Pirogov”, Sofia, Bulgaria; ^4^ Department of Rheumatology, Clinic of Rheumatology, University Hospital “St. Anna” Sofia, Medical University-Sofia, Sofia, Bulgaria, mu-sofia.bg; ^5^ Department of Informatics, New Bulgarian University, Sofia, Bulgaria, nbu.bg; ^6^ Medical Faculty, Medical University of Plovdiv, Plovdiv, Bulgaria, mu-plovdiv.bg; ^7^ Pediatric Department, University Multiprofessional Hospital for Active Treatment and Emergency Medicine “N. I. Pirogov”, Sofia, Bulgaria; ^8^ National Sports Academy, “Vassil Levski”, Sofia, Bulgaria; ^9^ Department of Healthcare, Faculty of Public Health, Medical University of Sofia, Sofia, Bulgaria, mu-sofia.bg

**Keywords:** inflammation, MIS-C, multisystem inflammatory syndrome in children, multivariant analysis, predictors of severity

## Abstract

**Background:**

Our goals were to find and grade children with multisystem inflammatory syndrome (MIS‐C) by clinical severity and the degree of different system involvement based on multivariate clinical laboratory data and identify the best predictors and scores for disease prognosis and risk stratification.

**Methods:**

We enrolled 51 patients with confirmed MIS‐C from a single center, and 34 general laboratory parameters and markers were included.

**Results:**

Using the *K*‐means clustering method on kernel principal component analysis (K‐PCA) projections, we identified that our MIS‐C patients could be separated into three clusters. Children belonging to Cluster 3 have the highest levels of ferritin, LDH, ASAT, ALAT, GGT, total bilirubin, direct bilirubin, azotemia, urea, and creatinine, followed by Cluster 2. In contrast, Cluster 1 showed the lowest levels, *p* < 0.05. Children belonging to Clusters 3 and 2 also needed inotropic support significantly more frequently than Cluster 1 (30% and 10% vs 0%, Fisher exact test *p* = 0.04). Furthermore, respiratory distress was found only in patients of Clusters 2 and 3 (*p* = 0.002). Regarding liver involvement, Clusters 2 and 3 more frequently had cholestasis (61% and 75% vs. 28%, *p* = 0.012), whereas Cluster 3 was more prominently characterized by an enlarged liver (44% vs. 0% and 5%, *p* = 0.004). Therefore, our clusters represent different grades, and liver and kidney involvement stages are graded by severity. From ROC curve analysis and several logistic regressions, we identified that age equal to or higher than 9 years old and ferritin levels higher than 470 ng/mL could help distinguish children with more severe kidney and liver involvement with a high probability (ROC AUC = 77*%*, *p* = 0.03 and ROC AUC = 75*%*, *p* = 0.04) approximately equal to the discriminatory potential of creatinine, urea, GGT and total bilirubin. Moreover, levels of LDH with a cutoff of 363 U/l could identify the children within Cluster 3 (ROC AUC = 80*%*, *p* = 0.004).

**Conclusions:**

LDH and ferritin, as well as age into consideration, could help the clinical assessment of the underlying severity.

**Keypoints**

MIS‐C patients’ stratification based on 34 clinical and laboratory parameters.

## 1. Background

The Coronavirus disease 2019 (COVID‐19) pandemic, which started in 2020, induced significant negative consequences and burdens for global health and the healthcare systems. A high percentage of the SARS‐CoV‐2 infection cases in children present with mild symptoms and no subsequent early complications [1]. However, in some instances SARS‐CoV‐2 can lead to multisystem inflammatory syndrome (MIS‐C) in children, an immune system overreaction leading to a hyperinflammatory state with life‐threatening potential. The Centers for Disease Control and Prevention′s (CDC) definition of MIS‐C is as follows: individuals younger than 21 years of age presenting with fever, laboratory evidence of inflammation, and evidence of clinically severe illness requiring hospitalization, with involvement of greater than or equal to two organs, with no alternative plausible diagnoses and a positive SARS‐CoV‐2 test (PCR, serology, and antigen) or COVID‐19 exposure within the preceding 4 weeks [2]. As of December 2022, the CDC reported over 9000 children with confirmed MIS‐C diagnosis, 76 of them with lethal outcomes due to complications [3]. The Royal College of Paediatrics and Child Health (RCPCH), the CDC, and the World Health Organization (WHO) have presented diagnostic criteria for MIS‐C [4]. Yet, no international criteria for predicting factors of the severity of the clinical progression of the disease are easily accessible or approved.

This is the fourth article by the collective (study group) focused on MIS‐C symptoms, manifestations, and clinical correlations [5–7]. The previous ones were focused on gastrointestinal and cardiac manifestations and complications only. In [5], our main subject is cardiovascular manifestations, whereas in [6], we focused on liver involvement. In [7], we investigated gastrointestinal involvement of this multisystem disorder. In contrast to previous studies, the current manuscript is centered specifically on clinical correlations between specific laboratory biomarkers and multiorgan system involvement, predictive modeling for disease severity, which has not been examined in previously published reports. Thus, although all four articles share a thematic connection (MIS‐C), their scientific objectives and hypotheses are distinct. We aim to enrich the MIS‐C topic in‐depth through this article series and provide a more comprehensive characterization of MIS‐C manifestations and severity.

According to our vast experience and the data available in the literature [3, 5, 6], children diagnosed under the MIS‐C umbrella term often tend to show a markedly diverse clinical presentation, system involvement, and varying degrees of symptom severity. Robust systemic hyperinflammation and immune system dysregulation provoked by previous COVID‐19 exposures are thought to be the culprit behind the heterogeneous multisystemic clinical presentation. Therefore, cluster analysis and identifying the underlying patterns and correlations between symptoms and laboratory paraclinical parameters could be potentially very important.

**Figure 1:**
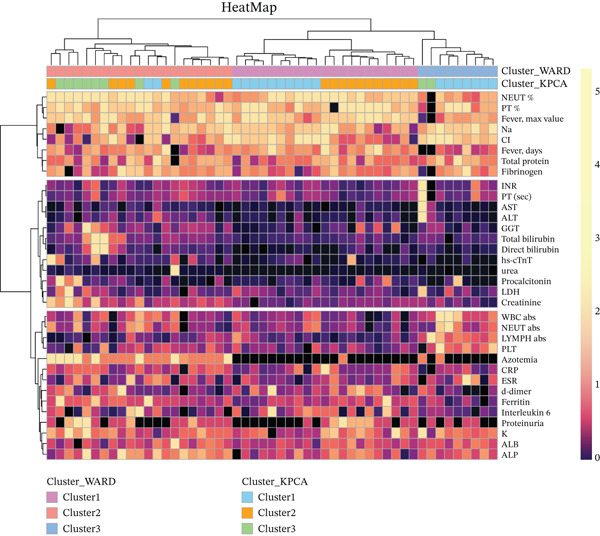
Heat map with annotation and dendrograms, displaying all study patients and their respective levels of the study parameters. All variables are min–max. A scaled and color scheme based on patient distribution is added in the upper right corner alongside an annotation description showing cluster affiliation by the WARD method and by K‐means on K‐PCA.

Our main study hypothesis is that MIS‐C patients group clusters that tend to differ significantly based on their paraclinical parameters linked with systemic inflammation and to correlate them with the degrees of clinical severity. Thus, our goals were firstly to find and grade MIS‐C children by clinical severity and the degree of different system involvement based on multivariate clinical laboratory data and secondly to identify the best laboratory markers and thirdly to propose score‐based approaches for disease prognosis and risk stratification. For study purposes, a total of 34 parameters and markers were included: hematological parameters—white blood cells (WBCs) absolute count, % and absolute count of neutrophils, platelets, and lymphocytes; inflammatory markers—C‐reactive protein (CRP), procalcitonin (PCT), ESR, interleukin‐6 (IL‐6), fibrinogen, and ferritin; levels of some electrolytes—Na, K, and Cl; liver function tests—aspartate aminotransferase (AST), alanine aminotransferase (ALT), gamma‐glutamyl transferase (GGT), total and direct bilirubin, and serum albumin and total protein levels; coagulation parameters—international normalized ratio (INR), prothrombin time (PT), and d‐dimer; kidney function parameters ‐ proteinuria, azotemia, urea, serum creatinine; and enzymes—LDH, alkaline phosphatase (ALP) and cardiac troponin; days with fever and maximum fever detected (Figure 1).

## 2. Material and Methods

### 2.1. Study Subjects and Design of the Study

All patients with confirmed MIS‐C (*N* = 51) from the pediatric department at the University Hospital “N. I. Pirogov,” (Sofia, Bulgaria) are enrolled prospectively in our study between the period of 25 November 2020 and 24 April 2021. Clinical assessment was in compliance with previous studies of the collective [7]. The pediatric department at University Hospital “N. I. Pirogov” is one of the main pediatric units in Sofia and in Bulgaria and served as a leading referral center for MIS‐C during the study period. All consecutive MIS‐C cases admitted to this department between 25 November 2020 and 24 April 2021 were prospectively enrolled. The present analysis was conducted on the same cohort of MIS‐C patients previously reported by the study group [5–7]. Although the study population is identical, each manuscript addresses different research questions and examines distinct clinical and laboratory variables. The current study focuses on finding multivariate clusters of disease severity and identifying score‐based approaches for disease prognosis and risk stratification, which has not been previously analyzed or reported.

Given the central role of this unit in MIS‐C care, our cohort likely represents a substantial proportion of the MIS‐C cases diagnosed nationally and can be considered reasonably representative of the Bulgarian MIS‐C population. Taking these two major considerations into account, we formulated our study design around the concept of case series study with prospective enrollment as a sampling method. Single‐centerness of our study should be taken as an additional advantage in our settings since we achieved to enroll 50% of all MIS‐C cases for the whole country population, thus making our data highly fair and representative for the Bulgarian MIS‐C population.

Predominantly, the children were male (*N* = 37, 72.5%) and over 5 years of age (*N* = 40, 78%). MIS‐C cohort′s mean age was 8.82 ± 4.16 years.

The sampling frame of our study was in line with the diagnostic criteria for MIS‐C formulated by the “RCPCH”, the CDC, and the WHO [4]. The inclusion criteria for the participants in the study were as follows:

1. Age under 18, fever > 38.0°C for ≥ 24 h (or subjective fever lasting ≥ 24 h), laboratory‐confirmed inflammation (assessed by elevated CRP, erythrocyte sedimentation rate (ESR), fibrinogen, PCT, d‐dimer, ferritin, LDH, IL‐6, higher neutrophils, low lymphocytes, and low albumin) and multisystem (> 2) organ involvement;
2. Exclusion of other diagnoses;
3. Confirmed recent or current infection with SARS‐CoV‐2 (RT‐PCR, antigen, or serological tests) or epidemiological data for exposure to the virus within the previous 4 weeks.

A negative nasopharyngeal rapid antigen test (except one, already described) and anti–SARS‐CoV‐2 seropositivity were present in all of the included patients. It was reported in a number of cases that a close family member with positive COVID‐19 was in contact with the patient in the previous 2 months before the inclusion in the study. A negative nasopharyngeal PCR result was initially present in 26 children; nevertheless, during hospitalization, the PCR result was positive in two of the mentioned patients.

### 2.2. Clinical Methods

All the children included in the study were admitted to the pediatric department of Sofia′s leading emergency hospital. Upon admission, each child received a thorough physical examination. The most notable symptoms observed at admission were abdominal pain and fever. Pediatric surgeons also assessed all children presenting with moderate to severe digestive symptoms (such as abdominal pain, vomiting, nausea, and diarrhea) both during the initial physical exam and throughout their hospital stay. Additionally, a comprehensive medical history was taken for each child at admission, detailing any concurrent conditions and any confirmed or suspected COVID‐19 infections within the past 2 months, or close contact with a confirmed COVID‐19 case within the family.

The general laboratory evaluation comprised of the following blood tests: complete blood count, ESR, inflammatory markers CRP, PCT, ferritin, AST, ALT, GGT, LDH, ALP, total serum proteins and albumin, creatinine and urea, total and direct bilirubin, and coagulation tests: INR, PT, d‐dimer, and fibrinogen. The hematological and biochemical indexes observed were interpreted based on age and sex, with the upper and lower limits of normal ranges corresponding to established pediatric reference specific reference ranges (see Table S1).

Additionally, serological evaluation for anti–SARS‐CoV‐2 antibodies was conducted using various methods in 50 children. IL‐6 levels were assessed in 32 children. A certified immunological laboratory conducted the serological anti‐SARS evaluation and IL‐6 assessment using Elecsys by Roche Diagnostics.

### 2.3. Statistical Analysis

For multivariate analysis and machine learning purposes, all variables were scaled using the min–max scaling technique (“MinMaxScaler”, scikit‐learn, Python). Agglomerative hierarchical and K‐means cluster analyses, kernel principal component analysis (K‐PCA), uniform manifold approximation and projection for dimension reduction were conducted in Jupyter Notebook (Python). Different metrics for internal cluster validation were estimated in Jupyter Notebooks (Python). Data visualizations were produced in RStudio (R programming language) and Hyper Notebook (Python). All other statistical methods (Mann–Whitney *U* tests, Kruskal–Wallis, or chi‐square tests) were conducted using licensed SPSS Version 29, *α* was set to 5%.

Clustering robustness and geometry checks. To assess sensitivity to K‐means geometry, we repeated K‐means (*n* = 100 starts) on the first four to six K‐PCA components; compared labels with Ward′s hierarchical solution; ran K‐medoids (PAM) and full‐covariance GMM; computed Silhouette and Calinski–Harabasz (CH) indices; examined per‐cluster covariance traces/eigen‐ratios in K‐PCA space; and visualized UMAP projections. Optimal cluster numbers were determined using multiple criteria, including Silhouette and CH scores (Table S2). The robustness of the clustering was confirmed through 200 resamples, as shown in the stability boxplots (Figure S1) and subsampling metrics (Table S3). Across methods, a three‐cluster solution was recovered with stable memberships and identical clinical gradients (ferritin, LDH, bilirubin, creatinine, and urea; inotrope use; respiratory distress), indicating that results are not driven by a spherical/equal‐size assumption.

## 3. Results

### 3.1. Social Demographics and Clinical Presentation

Fifty one children meeting the CDC′s MIS‐C diagnostic criteria were consecutively enrolled in the study. Detailed data regarding the demographics, important clinical parameters, and COVID‐19 exposure status can also be found in the previous articles from our study group [5, 6].

### 3.2. Cluster Identification

Firstly, the predictive potential of the study parameters that differed widely across the three clusters is summarized in Table 2 the continuous variables were min–max scaled. In order to better capture the underlying structure in our data, we conducted K‐PCA as a dimensionality reduction method. The concept behind K‐PCA is to perform a dimensionality reduction method for linearly nonseparable cases and use the first kernel principal components for cluster analysis to reduce the “noise” in the data and to extract the most important features of the underlying data structure for further cluster analysis.

We conducted a comprehensive selection and stability analysis for *k* = 2, 3, 4, 5, 6. Beyond Silhouette and CH, we report Davies–Bouldin (DB) and the gap statistic, as well as model‐based evidence via Gaussian mixture models (GMMs) BIC and a shape‐robust alternative (K‐medoids/PAM) (Table S2). We further assessed stability under subsampling (80% resamples, 200 runs), feature‐space perturbations (Gaussian jitter), and multiple initializations (100 starts), summarizing Adjusted Rand Index (ARI)/Normalized Mutual Information (NMI) and cluster‐wise Jaccard persistence, as shown in the stability boxplots (Figure S1) and subsampling metrics (Table S3). Across criteria, *k* = 3 is consistently supported: it maximizes/near‐maximizes Silhouette and CH, minimizes DB, is picked by lowest GMM‐BIC, and shows high stability (mean ARI/NMI across resamples) relative to *k* = 2, 4, 5, 6. Importantly, the clinical gradients (e.g., ferritin, LDH, bilirubin/creatinine/urea; inotrope use; respiratory distress) are monotone across the three groups and reproducible under PAM and full‐covariance GMM, indicating the result is not method‐specific. Median values for key variables such as ferritin, LDH, and liver enzymes remained consistent across different clustering algorithms (Table S4). To address residual concerns about size/shape, we added K‐medoids (PAM) and GMMs with unequal covariances (full‐GMM) as sensitivity analyses. Both alternatives reproduced the same three‐group structure with ARI 0.72–0.78 versus K‐means and the same clinical severity gradients (liver/kidney markers, inotropes, respiratory distress), supporting that our findings do not depend on a spherical–equal‐size assumption.

Independently of K‐means, Ward′s hierarchical clustering (Euclidean) also selected three clusters by internal indices (Silhouette and CH), supporting a compact‐cluster structure (Figure 1). On UMAP projections, the K‐means‐on‐K‐PCA solution followed the global/local relationships better than Ward′s solution (Figure 2); clusters appeared compact with limited overlap, which is consistent with K‐means′ preference for near‐spherical groups in the transformed space.

Figure 2Scatter plots display the distribution of the patients using both cluster methods: (a) and (b) on the UMAP Dimension 1 and UMAP Dimension 2.

**Figure:**
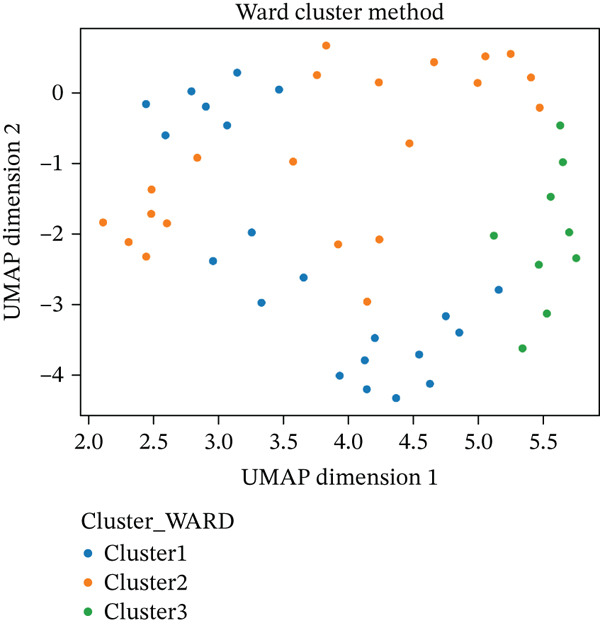
(a)

**Figure:**
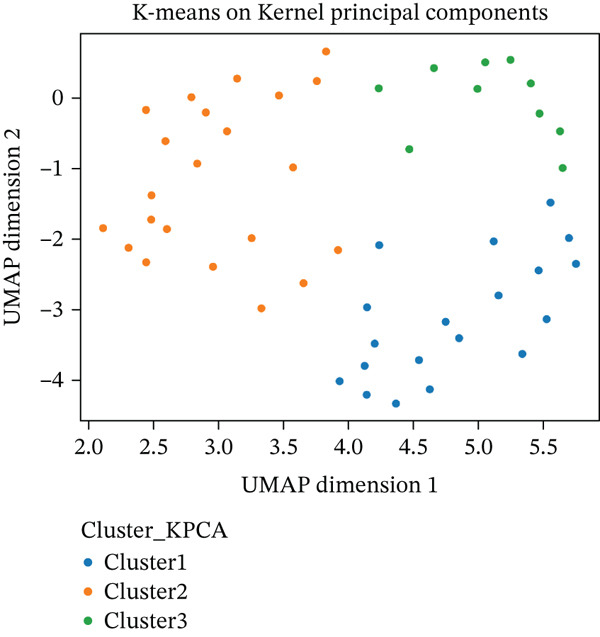
(b)

In sum, by scaling, mapping to a K‐PCA space, and cross‐validating structure with Ward, UMAP, and two non–K‐means algorithms, we showed that our clinical data behave close enough to K‐means′ favorable regime and, crucially, that our clinical conclusions are robust to clustering method. Across multiple clustering algorithms (K‐means on K‐PCA, Ward′s hierarchical clustering, K‐medoids/PAM, and full‐covariance GMM) and a range of internal validation metrics, the three‐cluster solution was consistently supported and reproduced, with stable clinical gradients in liver and kidney involvement, inotropic support, and respiratory distress.

### 3.3. Multivariate Clusters Comparison and Interpretation

By this moment, we identified that our MIS‐C patients could be separated into three clusters. As mentioned in the introduction section, our hypothesis was to evaluate the clinical significance of the underlying structure in our data. In line with our study goal, conducting multiple Kruskal–Wallis analyses were done to identify the study parameters that differ across the three formed clusters. Significant differences between the three clusters were found regarding the following parameters: age, ferritin, LDH, ASAT, ALAT, GGT, total bilirubin, direct bilirubin, azotemia, urea, and creatinine; detailed information alongside the *p* values are listed on Table 1 and Figure 3a), all other study markers did not show significant difference across the clusters. The variables were also compared based on their relative importance for discriminating the tree cluster using decision tree analysis (Figure 3b).

**Table 1:** Significant differences in study parameters across the three clusters. Data are presented as median (min–max) or mean ± std.deviation.

Variable	Cluster membership			
	Cluster 1 (*N* = 19)	Cluster 2 (*N* = 22)	Cluster 3 (*N* = 10)	Difference between clusters, *p*
Male/female ratio	14/5	15/7	8/2	*p* = 0.78
Age (years)	6.24 ± 3.8	10.26 ± 3.3	10.5 ± 4.1	*p* = 0.02^∗^
Ferritin	268.8 (67.5–1442.6)	564 (285.7–1550.8)	626.2 (235–1500)	*p* = 0.008^∗∗^
LDH (U/I)	283.5 (217–549)	300 (186–457)	431 (251–1159)	*p* = 0.014^∗^
ASAT (U/L)	34.5 (22–255)	44.5 (12–136)	102 (16–1168)	*p* = 0.048^∗^
ALAT (U/L)	21 (11–279)	36.5 (12–149)	85.5 (7–634)	*p* = 0.056^∗^
GGT (U/L)	14.5 (3–86)	53 (5–212)	99 (35–416)	*p* = 0.002^∗∗^
Total bilirubin (*μ*mol/L)	6.7 (0.1–16.3)	10.9 (2.8–45.4)	22.4 (12.2–103.6)	*p* = 0.001^∗^
Direct bilirubin (*μ*mol/L)	1.55 (0.5–5.2)	5 (1.2–24.7)	18.35 (4.9–71.8)	*p* = 0.001^∗∗^
Azotemia	0 (0–1)	1 (0–2)	2 (0–2)	*p* = 0.001^∗∗^
Urea (mmol/L)	3.4 (1.2–8.2)	5.7 (3.4–13.2)	11.2 (3–310)	*p* = 0.001^∗∗^
Creatinine (*μ*mol/l)	45 (26.82–66)	61 (42–149)	93.5 (44–182)	*p* = 0.001^∗∗^

^∗^ *p* < 0.05

^∗∗^ *p* < 0.01

Figure 3Data structure across MIS‐C patients: (a) Radar plot displaying the significant differences across the cluster, study parameters are min–max scaled and depicted on the radar plot axes; (b) heat map of the Spearman′s correlations between the significant differences across the three clusters. The color scheme shows the magnitude of the correlation coefficient and the corresponding *p* values are also depicted on the heat map.

**Figure:**
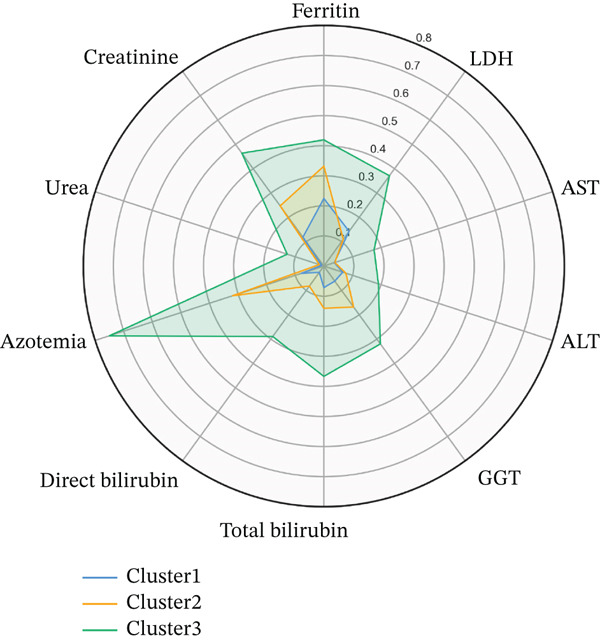
(a)

**Figure:**
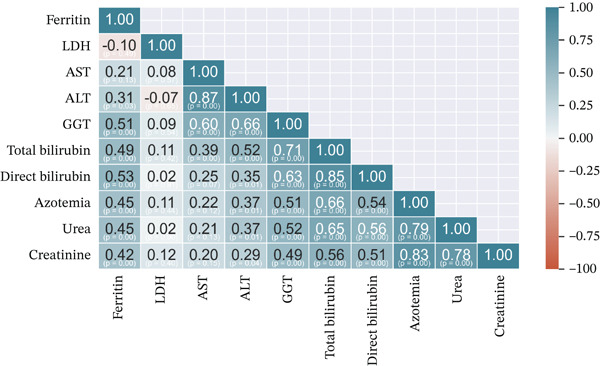
(b)

Furthermore, children belonging to Cluster 3 have the highest levels of ferritin, LDH, ASAT, ALAT, GGT, total bilirubin, direct bilirubin, azotemia, urea, and creatinine, followed by Cluster 2, whereas Cluster 1 show the lowest levels, Table 2. Important to note is that the clusters do not intersect on the radar plot and have similar shapes. The interpretation of those two findings could be that our clusters represent three different profiles of the severity of all other systems described by the laboratory parameters simultaneously, and they do not affect one system more than another when compared. That hypothesis was easily proven to be true by comparison between the correlation coefficients across the different clusters. Fisher′s *r*‐to‐*z* transformation was applied for correlation coefficients, and no significant differences were found. Hence, they follow the same correlational structure as the laboratory parameters. The correlational structure for all clusters is depicted in Figure 3c.

**Table 2:** ROC curve characteristics of study parameters for predicting cluster membership.

	Clusters 2 and 3 versus Cluster 1						
	*ROC AUC*	*p*	*Cutoff*	*Sensitivity*	*Specificity*	*OR*	*Predicted probability*
Age	*0.77*	*p* = 0.03^∗^	*8.5*	*76%*	*87%*	*9.5*	*85%*
CRP	*0.66*	*p* = 0.19	*NA*	*NA*	*NA*	*NA*	*NA*
Ferritin	*0.75*	*p* = 0.04^∗^	*470*	*77%*	*67%*	*13*	*84%*
LDH	*0.55*	*p* = 0.65	*NA*	*NA*	*NA*	*NA*	*NA*
T. bilirubin	*0.8*	*p* = 0.016^∗^	*9.5*	*70%*	*88%*	*9*	*85%*
Urea	*0.87*	*p* = 0.001^∗∗^	*4.6*	*70%*	*90%*	*15*	*86%*
Creatinine	*0.87*	*p* = 0.002^∗∗^	*50*	*87%*	*80%*	*20*	*87%*
	Cluster 3 versus Clusters 1 and 2						
	*ROC AUC*	*p*	*Cutoff*	*Sensitivity*	*Specificity*	*OR*	*Predicted probability*
Age	*0.63*	*p* = 0.09	*NA*	*NA*	*NA*	*NA*	*NA*
CRP	*0.62*	*p* = 0.24	*NA*	*NA*	*NA*	*NA*	*NA*
Ferritin	*0.7*	*p* = 0.162	*NA*	*NA*	*NA*	*NA*	*NA*
LDH	*0.8*	*p* = 0.004^∗^	*363.5*	*70%*	*87%*	*11*	*53%*
T. bilirubin	*0.91*	*p* = 0.001^∗∗^	*16.5*	*85%*	*83%*	*20*	*60%*
Urea	*0.800*	*p* = 0.040^∗^	*5.6*	*90%*	*72%*	*22*	*45%*
Creatinine	*0.83*	*p* = 0.001^∗∗^	*66.5*	*80%*	*80%*	*16*	*50%*

^∗^ *p* < 0.05

^∗∗^ *p* < 0.01

Children belonging to Clusters 3 and 2 also needed inotropic support significantly more frequently than Cluster 1 (30% and 10% vs. 0%, Fisher exact test *p* = 0.04). Furthermore, respiratory distress was found only in patients of Clusters 2 and 3 (*p* = 0.002). Regarding liver involvement, Clusters 2 and 3 more frequently had cholestasis (61% and 75% vs. 28%, *p* = 0.012), whereas Cluster 3 was more prominently characterized by enlarged liver (44% vs. 0% and 5%, *p* = 0.004).

### 3.4. Predictive Potential of Study Markers

**Figure 4:**
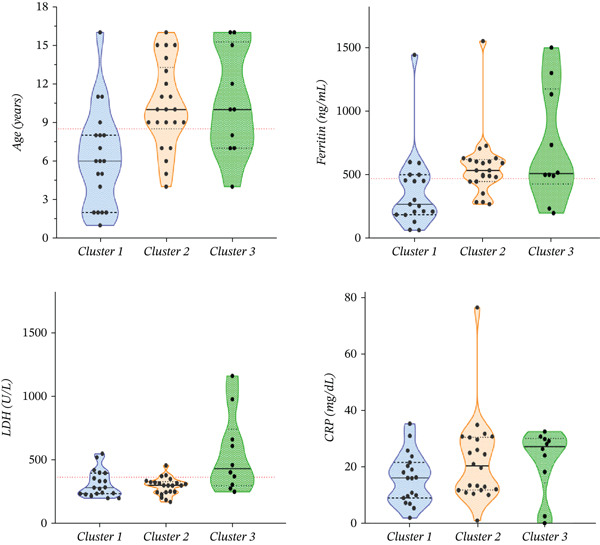
Box plots show age (a), ferritin (b), LDH (c), and CRP (d) distribution across the three clusters. The red dotted line depicts the cutoffs found by the ROC curve analysis summarized in Table 2.

Since the three clusters clearly separated the MIS‐C patients by severity accordingly and important clinical and laboratory correlations were found, we thought that of significant importance for pediatricians would be to explore the predictive capacity of different study markers in order to assign particular patients to the cluster. Receiver operating characteristic (ROC) curve analysis and several logistic regressions were performed, and the predictive potential of the study parameters that differed widely across the three clusters is summarized in Table 2. The distribution of age, ferritin, LDH, and CRP along with the cutoffs found by the ROC curve analysis across the three clusters are depicted on Figures 4a, 4b, 4c, and 4d.

ROC analysis was conducted to evaluate the discriminatory power of selected laboratory and clinical parameters for predicting cluster membership among patients with systemic manifestations. The comparison of Clusters 2 and 3 versus Cluster 1 demonstrated that age, ferritin, total bilirubin, urea, and creatinine were significant predictors of more severe or systemic phenotypes. Among them, urea and creatinine showed the highest area under the curve (AUC = 0.87, *p* = 0.001–0.002), reflecting strong discriminative performance with sensitivities of 70%–87% and specificities of 80%–90%. Total bilirubin (AUC = 0.80, *p* = 0.016) and ferritin (AUC = 0.75, *p* = 0.04) also demonstrated good diagnostic accuracy, with optimal cutoff values of 9.5 and 470 ng/mL, respectively. Age above 8.5 years (a marker distinguishing pediatric from adult clusters) yielded a sensitivity of 76% and specificity of 87% (*p* = 0.03). In contrast, CRP and LDH did not reach statistical significance for this comparison.

When comparing Cluster 3 versus Clusters 1 and 2, representing the most severe systemic pattern, total bilirubin, creatinine, urea, and LDH retained their predictive power. The strongest predictors were total bilirubin (AUC = 0.91, p = 0.001) and creatinine (AUC = 0.83, *p* = 0.001), with cutoff values of 16.5 and 66.5 *μ*mol/L, respectively. These parameters exhibited high sensitivity (80%–85%) and specificity (80%–83%), with odds ratios ranging from 16 to 20. Urea (AUC = 0.80, *p* = 0.04) and LDH (AUC = 0.80, *p* = 0.004) also contributed significantly, indicating systemic metabolic involvement and ongoing tissue injury in this subgroup. Collectively, these results support the use of renal and hepatic function markers as key discriminants of systemic involvement phenotypes, outperforming traditional inflammatory markers such as CRP.

## 4. Discussion

Our multivariate analyses′ results achieved our predetermined goals and revealed an underlying structure and pattern in our cohort of MIS‐C patients. We identified three major clusters with significant differences in levels of laboratory and clinical parameters that are intricately connected to the liver and kidney functions: the highest levels of AST, ALT, GGT, total and direct bilirubin, serum creatinine, urea, LDH, and ferritin were found among children in Cluster 3, followed by Cluster 2. Moreover, Clusters 2 and 3 displayed more frequent signs of respiratory distress, cholelithiasis, enlarged liver assessed by ultrasonography, and also the need for inotropic support. Therefore, our clusters represent different grades, and liver and kidney involvement stages are graded by severity. Worth paying attention to is the strong correlation between the involvement of liver and kidney dysfunction that defines the observed patterns and denotes the important mechanisms of COVID‐19 infection–induced injuries that target both axes largely and shape the clinical symptoms and paraclinical parameters of MIS‐C regarding adult patients. Thus, achieving an in‐depth understanding of MIS‐C renders the clarification of the intimate mechanism of COVID‐induced injuries and tropism to liver and kidney axes in pediatric patients who develop multi‐inflammatory syndrome.

As the COVID‐19 pandemic progressed, serum ferritin became one of the inflammatory biomarkers of COVID‐19 infection. In a meta‐analysis in 2022 in adult patients with COVID‐19, patients with severe disease (requiring ICU stay and mechanical ventilation, as well as showing kidney involvement) and subsequently negative outcomes presented with higher levels of serum ferritin in comparison with the ones with moderate to mild disease [8]. Serum ferritin can be influenced by age and comorbidities; however, the latter is rare in most cases in children. Ferritin is an inflammatory marker, an acute phase reactant that responds to the human body′s acute and chronic inflammatory processes [9]. Ferritin is a cytosolic protein, and under inflammatory conditions, with apoptotic and pyroptotic cellular death, it can be released in the extracellular space. For that reason, its increased serum levels in COVID‐19 denote considerable cellular damage. Increased LDH levels in the context of an ongoing COVID‐19 infection or its subsequent complications are another common serum marker that indicates cellular and tissue destruction since it is a predominantly cytoplasmic (oxidoreductase) enzyme. Furthermore, increased LDH levels can also trigger proinflammatory cytokine production via various Toll‐like receptor (TLR) ligations.

The ROC analysis underscores that biochemical indicators of organ function, rather than classical inflammatory markers, provide better discrimination between clinical phenotypes of systemic involvement [6]. Elevated urea, creatinine, and bilirubin levels likely reflect systemic inflammation with endothelial and mitochondrial dysfunction extending beyond the respiratory tract. The relatively lower performance of CRP and ferritin suggests that while these parameters capture acute‐phase activity, they lack specificity for chronic postviral immune dysregulation and multiorgan involvement. Importantly, the trade‐off between sensitivity and specificity should inform clinical interpretation. A creatinine threshold of 50–66 *μ*mol/L and a urea threshold of 4.6–5.6 mmol/L provided balanced sensitivity (~80%) and specificity (~80%–90%), allowing reliable identification of patients at higher risk of systemic manifestations while minimizing false positives. In contrast, bilirubin′s higher specificity (83%–88%) but moderate sensitivity (70%–85%) makes it more useful as a confirmatory marker rather than a screening tool. These differences suggest that a multimarker approach, integrating renal (urea and creatinine), hepatic (bilirubin), and metabolic (LDH) parameters, would enhance clinical accuracy compared with any single test.

Despite the difference in the definition of acute kidney injury (AKI) used between centers, a systematic review of 662 children with MIS‐C reported that around 16% of them progressed to develop AKI [10]. In a cohort of 166 children with MIS‐C and COVID‐19 infection, published in 2021, 11.8% developed AKI, 18.2% of which in MIS‐C patients. All of the MIS‐C patients who developed AKI presented with symptoms from the gastrointestinal system beforehand. Additionally, an association with lower albumin levels and higher WBC count was observed. Systolic dysfunction was reported in all of their AKI patients, which can be connected to the prerenal cause of AKI development in MIS‐C pathogenesis, mainly renal hypoperfusion [11]. AKI is a serious complication of MIS‐C syndrome. The odds of mortality in children with AKI and MIS‐C are higher than in children without it, as stated in a meta‐analysis published in 2023 [12]. Therefore, creatinine, urea, and urine output could be important markers of MIS‐C severity.

The mechanisms that lead to AKI in MIS‐C could be linked to immune hyperactivity and cytokine production and a prerenal injury due to hypovolemia, endothelial damage, or usage of nephrotoxic drugs [13], and virus‐induced tubular damage. Diorio et al. hypothesized that complement activation leads to subsequent vascular damage since elevated levels of sC5b9 were independent predictors of renal injury [14]. Deep et al. found a significant statistical correlation between hyperferritinemia and AKI incidence [13].

The involvement of the gastrointestinal tract is substantial in MIS‐C—in an observational study of 51 patients with MIS‐C in 2023, around 90% had gastrointestinal manifestation [14]. As for the involvement of the hepatobiliary system, SARS–CoV‐2 mechanisms of liver damage could be related to the attachment of the spike protein of the virus to the ACE2 receptor (a Type I integral membrane protein, modulating blood pressure via negatively regulating the renin–angiotensin system). Expression of the ACE2 receptor in the Type 2 alveolar epithelial cells, hepatocytes, and cholangiocytes allows viral entry and its cytopathic effects after that. Additionally, drugs used for symptomatic and COVID‐19 treatment that have liver metabolism can further contribute to cell toxicity. Hypoxia and cytokine storm are another set of factors during COVID‐19 infection associated with liver damage [6].

Liver injury associated with MIS‐C is immune‐mediated and is consequent to the immense synthesis and dispensation of a wide array of inflammatory cytokines (IL‐1*β*, IL‐1RA, IL‐6, IL‐8, IL‐10, IL‐17, IL‐18, IFN‐*γ*, and (TNF‐*α*) tumor necrosis factor *α*) leading to multiorgan damage. The pathogenesis of the liver involvement in MIS‐C shows resemblance to the alteration in the liver presenting during sepsis [6]. IL‐6 is one of the inflammatory markers associated with liver damage in acute COVID‐19 infection in adults [15]. By contrast, in a monocentric study in Italy, acute liver injury in children with MIS‐C was related to high levels of serum ferritin rather than IL‐6. [1]. What is more, elevated transaminases are significantly associated with MIS‐C′s clinical course and severity of presentation. In a single‐center observational study of 89 children with MIS‐C and COVID‐19 infection, approximately 50% of the patients presented with liver injury [6]. IL‐6 and CRP are early inflammatory markers, reflecting the magnitude of the initial virus‐induced inflammation. However, multisystem involvement and the severity of MIS‐C are consequences of both the intensity and the duration of the inflammatory process.

The key factor is the failure of immunosuppressive mechanisms to control excessive inflammation. IL‐6 and CRP do not directly indicate this failure. With this in mind, it becomes clear why clusters representing different degrees of multisystem involvement do not differ significantly in their IL‐6 and CRP levels. Also, it may be that initial inflammation is of the same magnitude across all clusters, but later malfunction of immunosuppressive mechanisms leads to different disease manifestations across all clusters.

Age is another variable that stood out and strongly correlated with our clusters. We found out that children of higher age (cutoff by the ROC curve analysis) developing MIS‐C are more prone to fall into Clusters 2 and 3, which are the more severe conditions, respectively. We hypothesize that immaturity could explain our findings since an immature immune system state is linked with increased immunosuppression and predomination of Th2 type immune response. However, in our study, we are limited only to finding correlations and interconnections, the explanations of which need extensive research of the precise mechanisms of COVID‐19‐induced injury causing immune hyperactivation.

Furthermore, in addition to identifying weak nodes and the central axes of COVID dysregulation in MIS‐C, we also focused on the predictive potential of LDH and ferritin to predict future system involvement in cases where symptoms are not yet present and to help develop objective criteria for clinical assessment of MIS‐C severity in routine practice. We identified that age equal to or higher than 9 years and ferritin levels higher than 470 ng/mL could help distinguish children with more severe kidney and liver involvement with a high probability (84%) approximately equal to the discriminatory potential of creatinine, urea, GGT, and total bilirubin. Moreover, levels of LDH with a cutoff of 363 U/L could identify the children within Cluster 3.

## 5. Limitations

Because MIS‐C is a rare condition, we included all available patient cases in our analysis, resulting in a relatively small cohort. A major limitation of our study is the lack of an external validation cohort, which prevents us from fully assessing or controlling for potential overfitting of the predictive algorithm. Consequently, the stability and reproducibility of the identified clusters in future patient populations remain uncertain. Validation using external datasets, ideally through collaboration with additional pediatric centers, will be an important direction for future research. Another limitation of our approach is that the clustering algorithm must be re‐executed whenever a new patient is added to the dataset, which restricts its feasibility for routine real‐time use in clinical settings. Future studies should develop user‐friendly algorithms or classification tools that enable automatic assignment of new patients to existing clusters without the need to repeat the entire clustering process.

## 6. Conclusions

LDH and ferritin, as well as age into consideration, could help the clinical assessment of the underlying severity. Interestingly, CRP, PCT, and IL‐6 could not distinguish the underlying clusters. Hence, they could not be interpreted as markers for systemic involvement and do not correlate with the central dysregulation of kidney and liver function axes in MIS‐C the way that LDH and ferritin do, which reaffirms the usefulness of LDH and ferritin as absolutely crucial predictors of disease severity. Furthermore, another benefit to note is that LDH and ferritin do not show a strong correlation with each other and thus could be applied as independent and complementary markers for severity stratification.

**Nomenclature**
AKI: Acute kidney injury
ALT: Alanine aminotransferase
ALP: Alkaline phosphatase
AST: Aspartate aminotransferase
CDC: Centers for Disease Control and Prevention
COVID‐19: Coronavirus cisease 2019
CRP: C‐reactive protein
GGT: Gamma‐glutamyl transferase
IL: Interleukin
INR: International normalized ratio
K‐PCA: Kernel principal component analysis
LDH: Lactate dehydrogenase
MIS‐C: Multisystem inflammatory syndrome in children
PCT: Procalcitonin
PT: Prothrombin time
RCPCH: Royal College of Paediatrics and Child Health
UMAP: Uniform Manifold Approximation and Projection for Dimension Reduction
WBCs: White blood cells
WHO: World Health Organization

## Author Contributions

Georgi Vasilev: conceptualization, methodology, formal analysis, writing—original draft preparation, writing—review and editing, visualization, supervision; Nadzhie Gorelyova: methodology, data curation, writing—original draft preparation, writing—review and editing; Russka Shumnalieva: data curation, writing—review and editing, supervision; Latchezar Tomov: methodology, formal analysis, writing—review and editing, visualization, supervision; Dayana Gandi Abousaid: methodology, writing—review and editing, supervision; Iren Tzotcheva: methodology, data curation, writing—review and editing; Snezhina Lazova: methodology, data curation, writing—original draft preparation, writing—review and editing, supervision; Tsvetelina Velikova: conceptualization, methodology, data curation, formal analysis, writing—original draft preparation, writing—review and editing, visualization, supervision, project administration, funding acquisition.

## Funding

This study was supported by the European Union‐Next Generation EU through the National Recovery and Resilience Plan of the Republic of Bulgaria (BG‐RRP‐2.004‐0008).

## Ethics Statement

This study was conducted in accordance with the Declaration of Helsinki and approved by the Ethics Committee of the University Hospital “N. I. Pirogov” under Protocol No 123‐20/23 December 2020. All parents signed their informed consent for the inclusion of their children in the study. Additionally, all children older than 12 years signed informed consent on their own before they participated in the study. This umbrella approval covers all analyses conducted and reported in previous articles by the study group [5–7] and the current manuscript.

## Conflicts of Interest

The authors declare no conflicts of interest.

## Supporting information


**Supporting Information** Additional supporting information can be found online in the Supporting Information section. Figure S1 provides boxplots illustrating cluster stability—including the Adjusted Rand Index (ARI), Normalized Mutual Information (NMI), and mean Jaccard index—across 200 resamples for different numbers of clusters ($k = 2\dots 6$). Table S1 details the hematological, inflammatory, coagulation, and biochemical study parameters alongside their respective pediatric reference ranges. Tables S2 and S3 present the cluster‐number criteria ($k = 2\dots 6$)  and stability metrics under subsampling/perturbation, respectively. Table S4 offers a comprehensive comparison of median clinical values to demonstrate the reproducibility of clinical gradients across K‐means, PAM, and GMM clustering methods.

## Data Availability

The data that support the findings of this study are available on request from the corresponding author. The data are not publicly available due to privacy or ethical restrictions.
