# Supplementary material for: Markers for the Severity of Multisystem Inflammatory Syndrome in Children: A Multivariate Analysis
Source: Int J Pediatr. 2026 Apr 10;2026:5409585. doi: 10.1155/ijpe/5409585 (PMC13067057; doi:10.1155/ijpe/5409585)
Supplement: Supplementary file 1 — Supporting Information Additional supporting information can be found online in the Supporting Information section. Figure S1 provides boxplots illustrating cluster stability—including the Adjusted Rand Index (ARI), Normalized Mutual Information (NMI), and mean Jaccard index—across 200 resamples for different numbers of clusters ($k = 2\dots 6$). Table S1 details the hematological, inflammatory, coagulation, and biochemical study parameters alongside their respective pediatric reference ranges. Tables S2 and S3 present the cluster‐number criteria ($k = 2\dots 6$) and stability metrics under subsampling/perturbation, respectively. Table S4 offers a comprehensive comparison of median clinical values to demonstrate the reproducibility of clinical gradients across K‐means, PAM, and GMM clustering methods. [file IJPE-2026-5409585-s001.docx]

**Supplementary Table 1.** Study parameters and their reference ranges. (8)

| **Study parameters** | |  |
| --- | --- | --- |
| **Hematological parameters** | | |
|  | WBC (10⁹ cells/L) | 1 month -2 years: 6.0-14.0;  2-9 years: 4.0-12.0;  10-17 years: 4.0-10 |
| **Inflammatory markers** | | |
|  | CRP (mg/L) | <1 year: 0.8-15.8  1-3 years: 0.5-11.2  4-10 years: 0.8-11.2  11-14 years: 0.6-8.1  15-18 years: 0.4-7.9 |
|  | Procalcitonine (ng/ml) | <0.5 |
|  | ESR (mm/hr) | Male – 0-15  Female – 0-20 |
|  | Interleukin-6 (pg/mL) | <17.4 |
|  | Fibrinogen (g/L) | 2.0-4.0 |
|  | Ferritin (µg/L) | 0-6 wееks: 0-400  7 weeks-1 year: 10-95  1-9 years: 10-60  10-18 years (male): 10-300  10-18 years (female): 10-70 |
| **Coagulation** | | |
|  | INR | <1.1 |
|  | D-dimer (ng/mL) | 1month-1year: 110-420  1-5 years: 90-530  6-10 years: 100-560  11-16 years: 160-390  >16 years: <500 |
|  | **Electrolites** |  |
|  | Na (mmol/L) | Infant – 133-144  Child – 134-143  Thereafter – 135-145 |
|  | K (mmol/L) | 1-6 months: 3.5-5.6  6 months-1 year: 3.5-6.1  >1 year: 3.3-4.6 |
|  | Cl (mmol/L) | 98-106 |
| **Liver function markers** | | |
|  | ASAT (U/L) | 1-3 years: 20-60  3-9 years: 15-50  10-15 years: 10-40  16-19 years (male): 15-45  16-19 years (female): 5-30 |
|  | ALAT (U/L) | <1 year: 12-45  1-19 years: 5-45 |
|  | GGT (U/L) | 4 months-10 years – 5-32  10-15 years: 5-24 |
|  | Direct bilirubin (µmol/l) | 1 month-adult: <5,1 |
|  | Total bilirubin (µmol/l) | 1 month-adult - <17 |
| **Kidney function parameters** | | |
|  | Urea nitrogen (mmol/L) | 1,8-6,4 |
|  | Creatinine (µmol/l) | < 4 years: 2,65-44,2  4-7 years: 2,65-52,2  7-10 years: 19,4-52,2  10-14 years: 27,4-77,8  >14 years: 44,2-93,7 |
|  | Protein in urine (mg/24h) | <150mg/24h |
| **Enzymes** | | |
|  | Troponin (ng/L) | <14 |
|  | LDH (U/L) | <1 year: 170-580  1-9 years: 150-500  10-19 years: 120-330 |
|  | Alkaline phosphatase (U/L) | 1-9 years: 145-420  10-11 years: 140-560  12-13 years: 105-495  14-15 years: 70-525  16-19 years: 50-260 |
|  | Albumin (g/dL) | <1 years: 19-49  1-3 years: 34-42  4-19 years: 35-56 |
|  | Total protein (g/L) | <1 years: 46-74  1-7 years: 61-79  8-12 years: 64-81  13-19 years: 66-82 |

**Supplementary Table 2.** Cluster-number criteria (k = 2…6).

| **k** | **Silhouette (mean ± sd)** | **Calinski–Harabasz** | **Davies–Bouldin** | **Gap** | **GMM BIC** | **PAM Silhouette** |
| --- | --- | --- | --- | --- | --- | --- |
| 2 | 0.363±0.09 | 33.69±2.43 | 0.55 | 0.167 | 5199 | 0.59 |
| 3 | 0.48±0.1 | 47.9±0.49 | 0.35 | 2.168 | 4304 | 0.66 |
| 4 | 0.283±0.02 | 31.74±1.97 | 0.38 | 1.276 | 4567 | 0.591 |
| 5 | 0.288±0.014 | 30.3±1.73 | 0.69 | 2.035 | 4334 | 0.664 |
| 6 | 0.286±0.017C | 30.73±1.92 | 0.86 | 1.974 | 4362 | 0.591 |

**Supplementary Table 3.** Stability under subsampling/perturbation (k = 2…6).

| **k** | **ARI (mean ± sd)** | **NMI (mean ± sd)** | **Mean Jaccard per cluster (min–max)** |
| --- | --- | --- | --- |
| 2 | 1±0.001 | 1±0.001 | mean 0.800, min=0.771, max=0.827 |
| 3 | 1±0.001 | 1±0.001 | mean 0.800, min=0.785, max=0.817 |
| 4 | 1±0.001 | 1±0.001 | mean 0.800, min=0.800, max=0.800 |
| 5 | 0.9±0.08 | 0.940.04 | mean 0.686, min=0.572, max=0.812 |
| 6 | 0.817±0.1 | 0.894±0.05 | mean 0.649, min=0.527, max=0.795 |

**Supplementary Table 4** Clinical gradients reproducibility (K-means vs PAM vs GMM**),** Comparison of Median Values

| Variable | K-Means | | | PAMM | | | GMM | | |  |
| --- | --- | --- | --- | --- | --- | --- | --- | --- | --- | --- |
|  | Cluster 1  (N= 19) | Cluster 2  (N=22) | Cluster 3  (N=10) | Cluster 1  (N= 18) | Cluster 2  (N=22) | Cluster 3  (N=11) | Cluster 1  (N= 16) | Cluster 2  (N=23) | Cluster 3  (N=12) | Kruskal–Wallis p |
| Ferritin | 268,8 | 564 | 626,2 | 288,8 | 554 | 616,2 | 257 | 575 | 637,2 | *p>0.05* |
| LDH (U/I) | 283,5 | 300 | 431 | 253,5 | 320 | 440 | 263,5 | 310 | 421 | *p>0.05* |
| ASAT (U/L) | 34,5 | 44,5 | 102 | 34,1 | 45.5 | 110 | 32,5 | 47,5 | 103 | *p>0.05* |
| ALAT (U/L) | 21 | 36,5 | 85,5 | 19 | 37 | 84 | 22.5 | 34 | 87 | *p>0.05* |
| GGT (U/L) | 14,5 | 53 | 99 | 16,5 | 50.2 | 101 | 12.8 | 55 | 98 | *p>0.05* |
| Total bilirubin (µmol/l) | 6,7 | 10,9 | 22,4 | 7 | 12 | 20.6 | 5 | 13 | 21.5 | *p>0.05* |
| Direct bilirubin (µmol/l) | 1,55 | 5 | 18,35 | 1,55 | 7 | 16,25 | 31,55 | 6 | 15,35 | *p>0.05* |
| Azotemia | 0 | 1 | 2 | 0 | 1 | 2 | 0 | 1 | 2 | *p>0.05* |
| Urea (mmol/L) | 3,4 | 5,7 | 11,2 | 3,4 | 5,7 | 11,2 | 3,4 | 5,7 | 11,2 | *p>0.05* |
| Creatinine (µmol/l) | 45 | 61 | 93,5 | 43 | 63 | 90 | 46 | 60 | 94,5 | *p>0.05* |


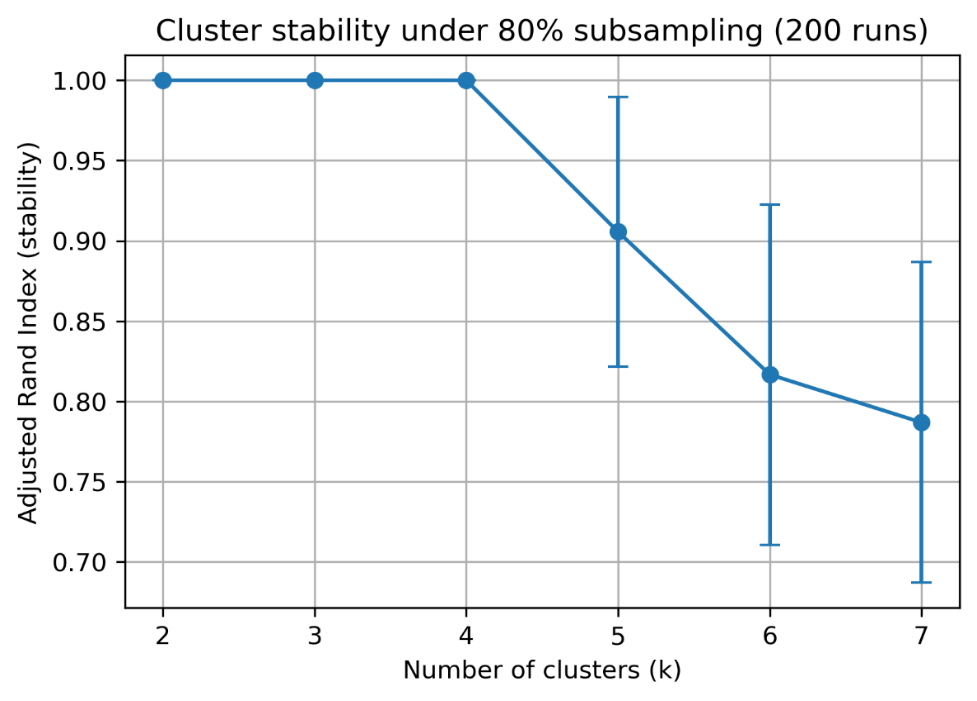
**Supplementary Figure 1**. Stability boxplots (ARI/NMI across 200 resamples) by $k$.

1. **
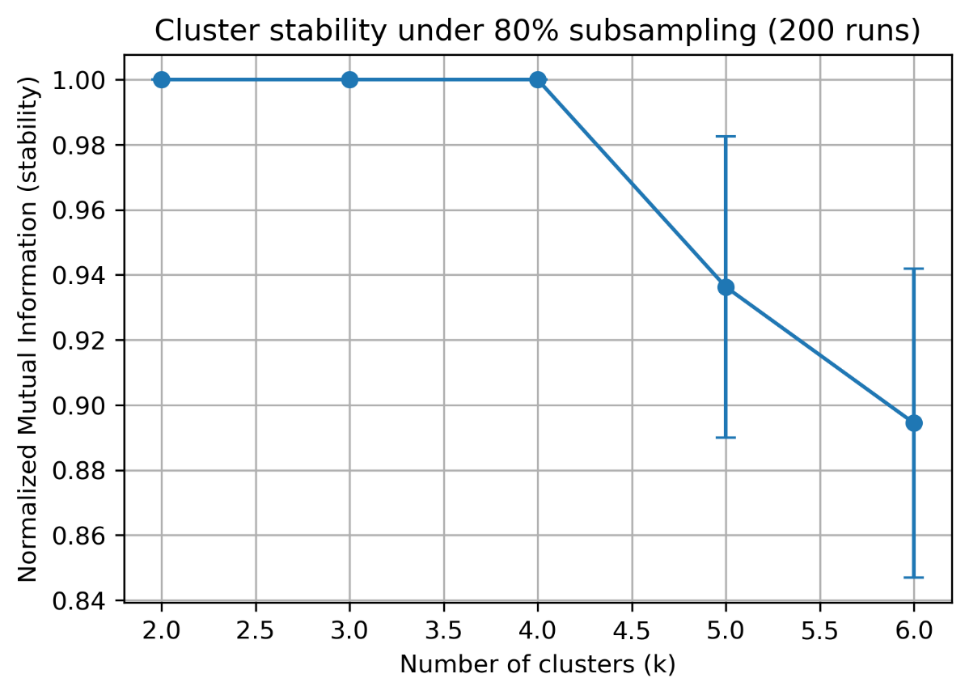
**Adjusted Rand Index across 6 clusters under 80% subsampling (200 runs)
2. Normalized Mutual Information across 6 clusters under 80% subsampling (200 runs)
3. **
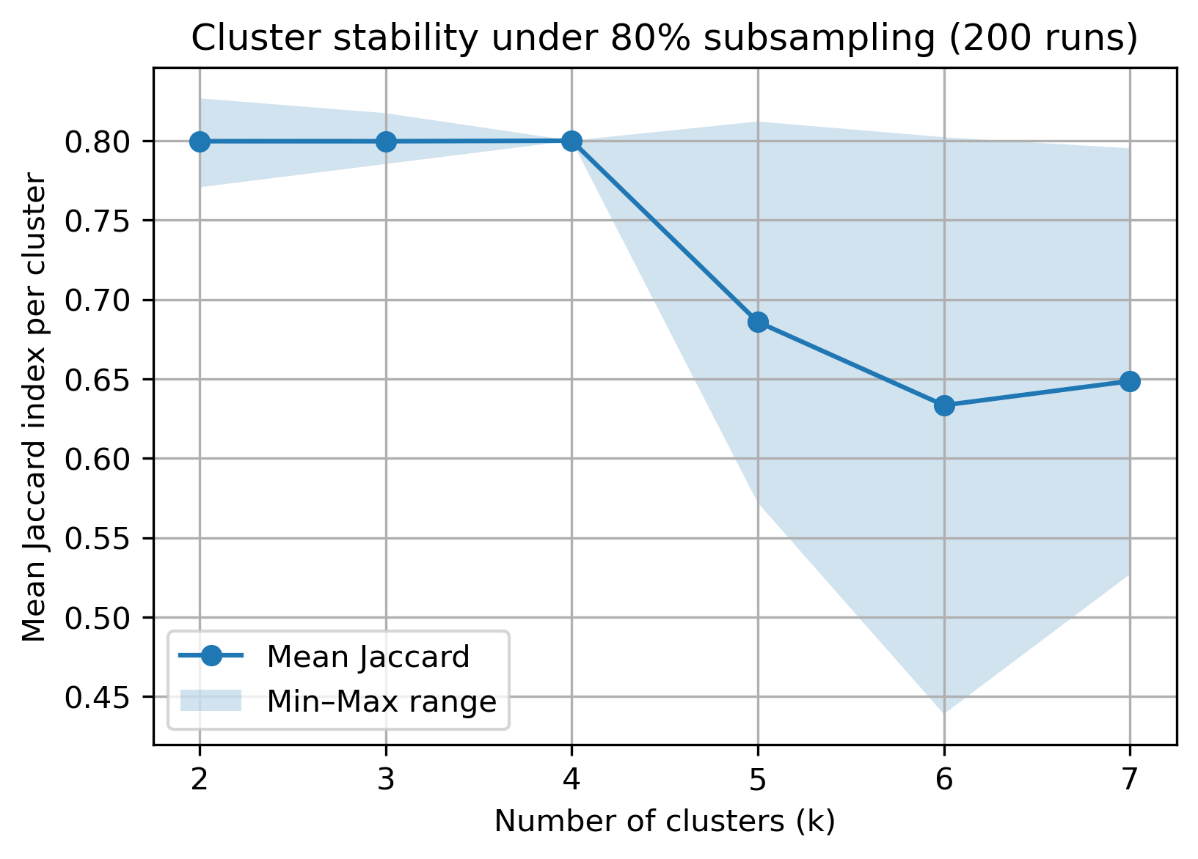
**Mean Jaccard Index across 6 clusters under 80% subsampling (200 runs)
